# Interdisciplinary Approach to Identify and Characterize COVID-19 Misinformation on Twitter: Mixed Methods Study

**DOI:** 10.2196/41134

**Published:** 2023-06-28

**Authors:** Iris Thiele Isip Tan, Jerome Cleofas, Geoffrey Solano, Jeanne Genevive Pillejera, Jasper Kyle Catapang

**Affiliations:** 1 Medical Informatics Unit College of Medicine University of the Philippines Manila Manila Philippines; 2 Behavioral Sciences Department De La Salle University Manila Philippines; 3 Mathematical and Computing Sciences Unit University of the Philippines Manila Manila Philippines; 4 English Language and Linguistics University of Birmingham Birmingham United Kingdom

**Keywords:** COVID-19, misinformation, natural language processing, Twitter, biterm topic modeling

## Abstract

**Background:**

Studying COVID-19 misinformation on Twitter presents methodological challenges. A computational approach can analyze large data sets, but it is limited when interpreting context. A qualitative approach allows for a deeper analysis of content, but it is labor-intensive and feasible only for smaller data sets.

**Objective:**

We aimed to identify and characterize tweets containing COVID-19 misinformation.

**Methods:**

Tweets geolocated to the Philippines (January 1 to March 21, 2020) containing the words *coronavirus, covid,* and *ncov* were mined using the GetOldTweets3 Python library. This primary corpus (N=12,631) was subjected to biterm topic modeling. Key informant interviews were conducted to elicit examples of COVID-19 misinformation and determine keywords. Using NVivo (QSR International) and a combination of word frequency and text search using key informant interview keywords, subcorpus A (n=5881) was constituted and manually coded to identify misinformation. Constant comparative, iterative, and consensual analyses were used to further characterize these tweets. Tweets containing key informant interview keywords were extracted from the primary corpus and processed to constitute subcorpus B (n=4634), of which 506 tweets were manually labeled as misinformation. This training set was subjected to natural language processing to identify tweets with misinformation in the primary corpus. These tweets were further manually coded to confirm labeling.

**Results:**

Biterm topic modeling of the primary corpus revealed the following topics: uncertainty, lawmaker’s response, safety measures, testing, loved ones, health standards, panic buying, tragedies other than COVID-19, economy, COVID-19 statistics, precautions, health measures, international issues, adherence to guidelines, and frontliners. These were categorized into 4 major topics: nature of COVID-19, contexts and consequences, people and agents of COVID-19, and COVID-19 prevention and management. Manual coding of subcorpus A identified 398 tweets with misinformation in the following formats: misleading content (n=179), satire and/or parody (n=77), false connection (n=53), conspiracy (n=47), and false context (n=42). The discursive strategies identified were humor (n=109), fear mongering (n=67), anger and disgust (n=59), political commentary (n=59), performing credibility (n=45), overpositivity (n=32), and marketing (n=27). Natural language processing identified 165 tweets with misinformation. However, a manual review showed that 69.7% (115/165) of tweets did not contain misinformation.

**Conclusions:**

An interdisciplinary approach was used to identify tweets with COVID-19 misinformation. Natural language processing mislabeled tweets, likely due to tweets written in Filipino or a combination of the Filipino and English languages. Identifying the formats and discursive strategies of tweets with misinformation required iterative, manual, and emergent coding by human coders with experiential and cultural knowledge of Twitter. An interdisciplinary team composed of experts in health, health informatics, social science, and computer science combined computational and qualitative methods to gain a better understanding of COVID-19 misinformation on Twitter.

## Introduction

Health misinformation is defined as a “health-related claim of fact that is currently false due to a lack of scientific evidence” [1]. Knowledge about COVID-19 evolved during the pandemic; hence, the inclusion of currency in this definition was particularly important. However, this definition does not include the intent and belief of the person sharing the health information. Although misinformation is false information that is shared inadvertently in the belief that it is true, disinformation is deliberately shared despite knowing that it is false and malinformation, although true, is used to inflict harm on others [2]. During a pandemic, too much information is available, a phenomenon the World Health Organization calls *the infodemic* [3]. This makes it difficult for the public to identify which health information is accurate and which is malinformation, misinformation, or disinformation. While not exclusively happening on the web, the infodemic is most evident on social media platforms, where even before the pandemic, health malinformation, misinformation, or disinformation could also be found.

While the availability of the Twitter API (application programming interface) and third-party platforms to mine tweets has made Twitter a popular platform for public health research, most of the published work has focused on high-income countries, analyzing tweets in the English language. A bibliometric study on health misinformation on social media found that half of the papers were contributed by the United States, where 80% of the most productive institutions were found [4]. This study contributes to the literature by analyzing geolocated tweets in the Philippines written in Filipino, English, or a combination of the Filipino and English languages.

Commonly used methodologies for public health Twitter research were sentiment mining and thematic analysis [5]. There is a need to integrate qualitative methods with computational approaches, as tweets are essentially social data that need to be interpreted in the context of social processes [6]. To understand the dynamics of how health misinformation spreads on social media, there is a need to examine not only the content but also the context and framing of health misinformation [7]. While previous literature on misinformation spanned separate disciplines [8], this study adopted an interdisciplinary approach, using a research team of experts in health, health informatics, social science, and computer science. Both computational and qualitative methods were used to answer the following research questions (RQs):

- RQ1: What is the prevalence of COVID-19 misinformation in tweets geolocated to the Philippines?
- RQ2: What are the topics, formats, and discursive strategies used in tweets with COVID-19 misinformation?

## Methods

### Overview

A convergent mixed methods design was used to identify and characterize tweets with COVID-19 misinformation. In the qualitative arm, key informant interviews and manual coding of tweets were completed. Tweets with misinformation were further characterized using constant comparative, iterative, and consensual analyses. In the quantitative arm, natural language processing (NLP) was used to identify tweets with misinformation. Integration was performed by correlating key informant interview findings with manually coded tweets. Tweets with misinformation identified using NLP were also manually coded to confirm labeling.

### Data Collection

Using the GetOldTweets library by Jefferson Henrique [9] in the Python programming language, tweets from January 1 to March 21, 2020, geolocated around the Philippine National Capital Region (NCR) and containing the words “coronavirus,” “covid,” and “ncov” were collected on June 15, 2020 [10].

The key informant interviews were conducted from November to December 2020 to elicit examples of COVID-19 misinformation within the period covered. Through snowball sampling, we recruited 10 key informants: pharmacists (n=2), a pharmacist-instructor, a pediatrician, a rheumatologist, a business manager, a nurse-instructor, a chief technology officer, a general practitioner, and a data manager. Semistructured interviews were conducted via videoconference or direct messaging after informed consent was obtained. The interview guide is presented in Multimedia Appendix 1. All the interviews were transcribed for analysis. The informants were also asked to share screenshots of tweets, Facebook (Meta Platforms Inc) posts, or Viber (Rakuten Inc) and Messenger (Meta Platforms Inc) messages that they considered misinformation. Keywords pertaining to COVID-19 misinformation (n=89; Multimedia Appendix 2) were derived from the interview transcripts and shared tweets, posts, and messages.

### Data Processing

The primary corpus was subjected to biterm topic modeling (BTM) to identify workable topic clusters for subcategory development. The topics identified by BTM were further reduced to clusters of keywords that were labeled based on the common meanings they espoused.

NVivo (QSR International) was used to query the corpus as follows: top 1000 most frequently occurring words, minimum length of 3 characters, and exact match only. This word list was first reduced by removing the following words and hashtags that were unlikely to discriminate between the tweets: *covid, ncov, coronavirus, #coronavirus, #covid, #ncov, #covid19, #covid2019, #coronavirusoutbreak, #covid19ph, #coronaviruspandemic.* Next, the stop words in the list were excluded. Words in the list were further excluded if they occurred in ≤100 tweets. To extract tweets likely to contain COVID-19–related misinformation, a text search was conducted on NVivo using the remaining words in the list and the key informant interview keywords. In this manner, subcorpus A was constituted with 5881 tweets.

Subcorpus A was manually coded to identify tweets with COVID-19 misinformation, informed by insights gained from the key informant interviews. Constant comparative analysis (CCA) was used to increase the trustworthiness of the study’s findings. It had three phases: (1) open coding, concepts found in both key informant interviews and tweets were coded and clustered into emergent categories; (2) axial coding, relationships among the categories were described; and (3) selective coding, categories were compared and aligned with existing theoretical propositions to generate core categories. Coding was performed using NVivo.

Iterative analysis was also used where the coding of the key informant interviews and the tweets informed each other. This is a repetitive process of going back and forth between interviews and mined tweets until the categories that best explained the phenomenon were identified. Finally, consensual qualitative analysis (CQA) was used. The interdisciplinary team met weekly to present analysis updates to arrive at a consensus on the codes and categories.

In a parallel analysis, the primary corpus was reduced to 4695 by including only those tweets containing keywords from the key informant interviews. This was then preprocessed to remove the following: URLs, words preceded by “#” or “@,” punctuation, English and Filipino stop words, words containing digits only, words with ≤2 characters, and tweets with ≤2 words. The data were then subjected to lowercasing and tokenization. The final training set, subcorpus B (n=4634), contained 506 tweets that were manually coded by 2 members of the team and labeled as “misinformation.” The Kullback-Leibler divergence for scoring both informativeness and phraseness to extract key phrases was used to identify more tweets with COVID-19 misinformation from the unlabeled tweets in the primary corpus. A more complete description of the NLP methodology is discussed elsewhere [11]. Tweets labeled as misinformation using NLP were further manually coded by health experts in the research team to confirm the labeling.

### Ethics Approval, Informed Consent, and Participation

The research protocol was approved by the University of the Philippines Manila Research Ethics Board (UPMREB 2020-629-01). In compliance with the Declaration of Helsinki, informed consent was obtained from key informants before the interview. Interview files and transcripts were stored in an encrypted cloud. Pseudonyms were used in data presentation to protect privacy. Only tweets from nonprivate accounts were collected. Account names, time stamps, and other traceable information were not included in the representative quotes used for the presentation of results. Representative tweets in Filipino or a mixture of Filipino and English were translated to English to render the tweets unsearchable on the web. This study adheres to the Standards for Reporting Qualitative Research guidelines [12].

## Results

### RQ1: What Is the Prevalence of COVID-19 Misinformation in Tweets Geolocated to the Philippines?

**Figure 1:**
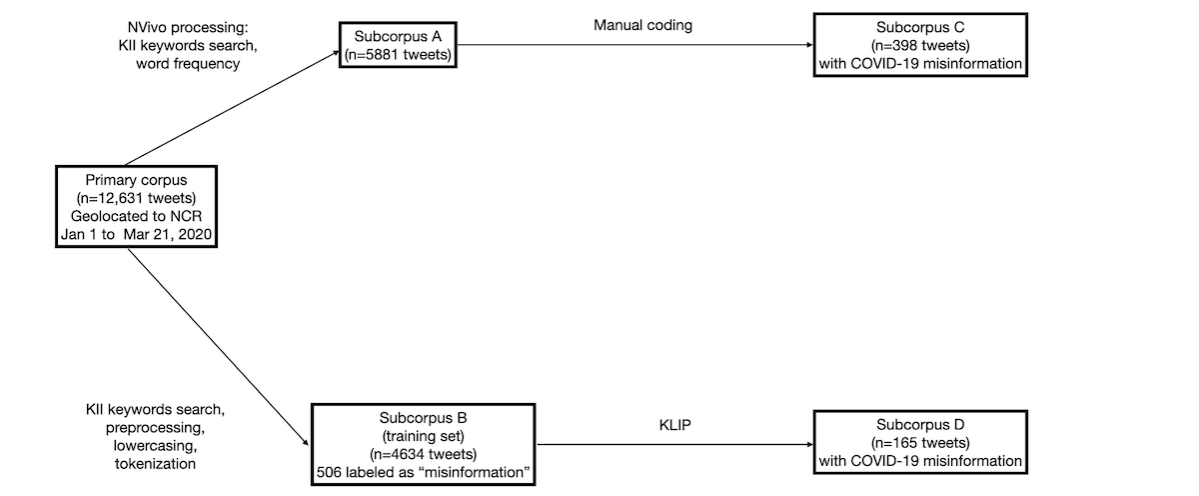
Process to identify tweets with COVID-19 misinformation. KII: key informant interview; KLIP: Kullback-Leibler scale for informativeness and phraseness; NCR: National Capital Region.

Figure 1 shows the parallel qualitative analysis and NLP, which identified tweets with COVID-19 misinformation in the primary corpus. Using manual coding, 6.8% (n=398) of tweets were found to have misinformation in subcorpus A. NLP labeled 165 tweets in subcorpus B as misinformation. Only 3 tweets with misinformation were identical to tweets found in subcorpus A. Manual coding by 2 health experts in the research team revealed that 69.7% (115/165) of tweets did not contain misinformation and were mislabeled using the computational approach.

### Characterizing the Primary Corpus

BTM identified 15 topic clusters from the primary corpus. Topic clusters and associated keywords are presented in Multimedia Appendix 3. Upon analysis of the common themes exhibited by the clusters, these were grouped into 4 topics presented in subsequent sections.

#### COVID-19 Prevention and Management

The topic emerged from six clusters: (1) safety measures, with keywords related to the recommended practices to prevent contracting the virus; (2) testing, with keywords indicating the process, results, and contexts of COVID-19 testing; (3) precautions, with keywords signifying the sense of threat and safety related to the virus; (4) health measures 1, with keywords related to general health promotion and sanitation activities; (5) social distancing, with keywords referring to policies related to decreasing social contact with others; and (6) health measures 2, which also includes general practices to protect oneself from contracting the virus.

#### Nature of COVID-19

This emerged from two topic clusters: (1) uncertainty, which includes keywords that signify terms related to the largely unknown facts about the virus during the beginning of the pandemic, and (2) COVID-19 cases, which includes keywords related to knowledge of confirmed cases and deaths of the disease during that time.

#### People/Agents of COVID-19

This refers to individual and collective actors during the early COVID-19 outbreak. This subcategory emerged from three topic clusters: (1) lawmaker’s response, which includes keywords related to legislative and other governmental entities; (2) international issues, which includes keywords related to countries and governments; and (3) frontliners, which includes keywords related to health care professionals and facilities.

#### Contexts and Consequences of COVID-19

This emerged from four topic clusters: (1) loved ones, with keywords referring to significant others and the need to protect them; (2) panic buying, with keywords related to the shortage of goods, such as mask and alcohol; (3) economy, with keywords indicating concerns about businesses and other economic activities that might be constrained by the pandemic; and (4) tragedies other than COVID-19, with keywords reflecting other crises that occurred in the country weeks before the entry of COVID-19, such as the Taal Volcano eruption, the death of Kobe Bryant, and the outbreak of other diseases.

### RQ2: What Are the Topics, Formats, and Discursive Strategies Used in Tweets With COVID-19 Misinformation?

#### Topics

Tweets with COVID-19 misinformation were classified under the 4 topics identified in the analysis of the primary corpus. Table 1 shows the topics with illustrative tweets corroborated by quotes from the key informant interviews.

**Table 1:** Topics with illustrative tweets of COVID-19 misinformation and quotes from key informant interviews (KIIs).

Topics	Sample tweets	Quotes from KIIs
1.1. COVID-19 prevention and management	“F**k you, COVID-19. The nurse told me to drink beer to avoid you. D**n, I'll drink this everyday. You won't get into my system. M*ron. I am drunk at the moment.”	“I think with regards to medicine, there were drugs already in the market that they wanted to study if it can be used for COVID. People think that since it’s being studied, it can be used. For example, hydroxycholoroquine, although it is used for moderate and severe cases, it’s not used for home remedies. Because of that, pharmacies’ inventory of hydroxychloroquine was depleted and a lot of lupus patients do not take the drug because they can’t buy it.” [Respondent G]
1.2. Nature of COVID-19	“COVID-19 is getting scarier. It’s already airborne. Are there safe spaces left? #COVID2019”	“...that COVID is only thriving in cold environments so we in the Philippines would not be affected because we are a tropical country.” [Respondent D]
1.3. People/agents of COVID-19	“And there are rumors that this COVID-19 is not actually from an animal but it was made by Chinese for biological warfare. I don’t know how true is this but it is a serious issue now that it affects the whole world.”	“There were a lot of conspiracy theories coming out. I read a lot about the different angles about how it came out and spread. One famous theory is that it’s actually manmade from a laboratory in Wuhan. Then, the CIA intervened because they want a sample of the virus. Then, it went as crazy as they wanted to use it as military technology to handle the issue in Hong Kong.” [Respondent A]
1.4. Context and consequences of COVID-19	“Experts told us we can contain COVID-19 and prevent deaths. But we did not listen. We still engaged in hoarding and panic buying. We gave in to panic and this poor lady got affected because of it.”	“There are hospitals reporting or was reportedly reporting to having COVID-positive patients so people don’t want to go to those hospitals to avoid getting infected when they go to the hospital. And then later, they find out that case isn’t COVID. They just have similar symptoms.” [Respondent D]

#### Formats

##### Overview

Further examination of subcorpus C showed common patterns in how the tweets with misinformation were constructed. This was also observed by the key informants. To aid in the reduction of the tweets to substantial categories depicting these patterns, a search for possible theoretical propositions to best explain the phenomenon was performed. The typology of misinformation or disinformation by Wardle and Derakhshan [2] was used to identify these formats in subcorpus C. These formats were also supported by insights gained from the interviews. The illustrative tweets are shown in Table 2.

**Table 2:** Topics, formats, and discursive strategies; frequency (n) and percentage (%) of occurrences; and sample tweets from subcorpus C (n=398).

Categories			Values, n (%)		Sample tweets
**Core 1: topics^a^**					
	1.1. COVID-19 prevention and management	191 (48)		“My wife said that due to the COVID-19 situation, there’s no more alcohol at home for sanitization purposes. But I have Martell Cordon Bleu.”	
	1.2. Nature of COVID-19	82 (20.6)		“COVID-19 is becoming scary. It is now airborne. Is there still anywhere safe? #COVID2019”	
	1.3. People/agents of COVID-19	71 (17.8)		“Kung Hei Fat Choi to our Chinese friends who spread the virus and disease. Happy Chinese New year! #coronavirus #ChineseNewYear #CNY2020 #YearoftheRat #ChineseNewYear2020”	
	1.4. Context and consequences of COVID-19	54 (13.6)		“NcOV is scary but I’m scarier. If our store closes, my salary goes to waste.”	
**Core 2: formats^b^**					
	2.1. Misleading content	179 (45.5)		“We Filipinos think we won’t get with CoVid 19, because we have strong resistance. It’s very hot here, we love to take a bath. In reality, we just don’t have test kits. It just arrived.”	
	2.2. Satire/parody	77 (19.2)		“It’s scary to do fieldwork. Don’t come close COVID. I still don’t have a boyfriend. Wait.”	
	2.3. False connection	53 (13.2)		“I think hand cream will sell well after this Covid. Our hands will dry up after all the hand washing and alcohol.”	
	2.4. False context	42 (10.4)		“It seems like a good time to drink. To avoid ncov hahaha!”	
	2.5. Conspiracies	47 (11.7)		“You only confirmed when other countries confirmed that they have COVID patients that came from here. So obvious.”	
**Core 3: discursive strategies^c^**					
	3.1. Humor	109 (27.4)		“Anti-COVID-19, 4 liquors for a stronger immune system.”	
	3.2. Fear mongering	67 (16.8)		“It’s scary to watch zombie movies especially now that there’s ncov.”	
	3.3. Political commentaries	59 (14.8)		“This covid was intentionally spread here to the Philippines. You know why? For the ABS-CBN franchise, so that the people will get mad at father Digong. Because he favors the Chinese, so this is their way of getting the people of the country angry with him. You don’t notice?”	
	3.4. Anger/disgust	59 (14.8)		“One fellow made a good point that this is not vague. They tried to hide the COVID-19 cases to avoid public panic, and now that it is time for the final reading of anti-citizen policies. They’re making noise on COVID to divert the attention of the masses. Like security of tenure.”	
	3.5. Performing credibility	45 (11.3)		“Correct. Actually I am not afraid of covid 19 but my kids are young so they have a weaker immune system. So I stay at home as much as I can.”	
	3.6. Overpositivity	32 (8.1)		“That’s a go.. Just bring lots of masks and vitamins to fight COVID.”	
	3.7. Marketing	27 (6.8)		“Because there are new COVID-19 cases now, it’s better to be safe and protected. These days, you don’t know who is sick. I’m selling Emergency Packs w/ Face Masks. There are 48 items inside. PM me for orders You can also check our FB (link to page selling packs)”	

^a^Subcategories under “topics” are based on emergent groupings of biterm topic modeling topic clusters.

^b^Subcategories under “formats” are partially based on the study by Wardle [13].

^c^Subcategories under “discursive strategies” are purely inductively emergent from our qualitative analysis of the tweets.

##### Misleading Content

This refers to tweets that frame the issue differently. In this regard, Respondent I (a data scientist) mentioned, “I was able to watch one video. It seemed like an altered video from a newsroom with two newscasters that appeared to discuss bananas as treatment.”

##### Satire and Parody

This refers to humorous content that can fool readers. However, this can still be perceived as true by those who cannot distinguish between factual and satirical content. This format was not mentioned by the informants but was found in abundance in subcorpus C.

##### False Connection

This refers to tweets in which the tweet is incongruous with the linked content. These posts are further described by Respondent F as content that comes with unrelated headlines. They mentioned, “sometimes, it would be a link to a website, like one that you would see on a tabloid. Very big headline.”

##### False Context

This refers to tweets with genuine content that are reframed differently. Respondent E, a data scientist, shares that posts like these were expected because there were so many experts sharing their expert opinions and journal articles on the web. “The public is not really used to reading this much information freely shared on the web,” he explained.

###### Conspiracy Theory

While tweets containing conspiracy theories may reasonably fall under the category of fabricated content by Wardle [13], fabricated content encompasses a wider variety of made-up posts, photos, or videos that may not necessarily pertain to conspiracy. We experienced difficulty imputing the intent of or proving fabrication. Tweets categorized as conspiracy theories have content that stipulates the pandemic as a secret plan by powerful people or groups [14]. Respondent A, a pharmacist, shared a conspiracy theory that spread early during the pandemic where the virus was said to have been “manmade from a laboratory in Wuhan.” This went further to state that the “CIA intervened because they want a sample of the virus...to use it as military technology to handle the issue in Hong Kong.”

Manipulated content and imposter content were not found in the corpus. Of the 5 formats identified in the corpus, the most common format was that of misleading content.

#### Discursive Strategies

##### Overview

From the team’s CQA of the tweets in subcorpus C, another logical pattern was observed to explain the composition of tweets with COVID-19 misinformation based on the explicit and implicit motives, moods, and/or persuasion styles they deploy and/or embody. Insights from key informant interviews also confirmed the discursive strategies that take form in tweets to appeal to an intended or unintended audience. Representative tweets are shown in Table 2.

###### Humor

This refers to tweets that use comedy, jokes, and funny antics and expletives to draw attention to untrue content. Some informants have explained that these overly positive and humorous contents can foster COVID-19 denial and acceptance of emerging unverified treatment and prevention measures.

###### Fear Mongering

This refers to tweets that use messaging that aims to elicit anxiety and fear, while also peddling misinformation. Most of the informants agreed that fear was a common facilitator of consumption and sharing of unreliable information on social media. Respondent I mentioned fear mongering posts that were related to the end of the world, stating that “among us the Evangelicals, there is a religious aspect to this pandemic. It is associated with the end of time...they post about prophecies and sermons, that will suggest that these are the signs of the end of times.”

###### Political Commentary

This refers to tweets that are critical or supportive of government responses regarding COVID-19, both directed toward the administration and the opposition, while including misinformation in the content. Respondent E further explained this, stating that “the discourse became antiDuterte, proDuterte [President of the Philippines].... These are all generating a lot of divisive content on social media.”

###### Anger and Disgust

This refers to tweets that deploy negative commentaries and emotions directed at people’s behavior early in the COVID-19 pandemic while also including misinformation in the content. In line with this, Respondent D (a nurse educator) shared, “I really think that everyone became emotional since COVID started...out of their emotions, they started caring for people without checking the correctness of their posts.”

###### Performing Credibility

This refers to tweets that deploy linguistic techniques to make nonfactual content appear science based. These include tweets that cite authorities to support their claims, such as physicians and other experts. In addition, the composition of these posts mimic legitimate news sources. In this regard, Respondent E explained, “the fake news looks as credible as the real news. It’s the same physician spreading it. It comes from a reputable publication.”

###### Overpositivity

This refers to tweets that deploy messages of hope, resilience, and motivation to overcome the pandemic; however, the content often disregards or downplays the gravity of the COVID-19 situation. In addition, these tweets include those that have religious and prayerful undertones.

###### Marketing

This refers to tweets that make use of advertising narratives to sell products and services, especially those that are claimed to be interventions against COVID-19. Respondent G (a pharmacist) shared about web-based posts that marketed supplements, stating that “there were those who were selling...they were marketing food supplements...they were posting pictures of how they could be contacted.”

On the basis of further analysis of the emergent core and subcategories of COVID-19 misinformation, formats were observed to adopt various discursive strategies and vice versa. Table 3 presents a matrix of the intersections of formats and discursive strategies through representative tweets.

**Table 3:** Matrix of the intersection of formats and discursive strategies of COVID-19 misinformation on Twitter.

Discursive strategies	Formats of COVID-19 misinformation				
	Misleading content	Satire/parody	False connection	Conspiracies	False context
Anger/disgust	“I'm so paranoid, darn it. My field work is in Binondo, there are a lot of Chinese there. Then, I go to the hospital everyday. I bought a lot of Vitamin C. There are no more masks, this Lysol is the last. Everyone is panic buying #MedRepLife #CoronaVirus”	“Face masks are a big help against nCOV but also to cover the mouths of gossipers!”	(Image showing empty shelves and expresses anger)	“S*n of a b*tch! You didn't learn from 2003 SARS which came from you. Now you created 2019-nCov! You animals!”	“Alcohol and face masks are not the solutions to avoid COVID-19. It's discipline.”
Fear mongering	“There’s so many Chinese at MOA sksksk Maybe there’s a carrier of the coronavirus among them? Huhu”	“F**k, I've been feverish yesterday and then I would read today that there is news that there is a confirmed case in BGC. My Lord!”	“There are two reports of covid-19 from people traveling to in Australia and Taiwan from the Philippines. Wtf is our government doing to address this. We’re going to literally be walking dead in no time.”	“I really don't believe that no one is infected with COVID in the Philippines yet. Our neighboring countries have so many infections already. Well, in fact there are so many Chinese here”	“I noticed that since covid started, there aren’t a lot of red ants anymore. This is really serious!”
Humor	(Image of a person using sanitary napkin as face mask)	“Sickness on Feb 14 is NCOV… N-No C-Cash O-On V-Valentines...”	“I think hand cream will sell well after this Covid. Our hands will dry up after all the handwashing and alcohol.”	—^a^	“You are only COVID-19, we are single. We are experts at Social Distancing and No Physical Contact.”
Marketing	Tweet links to an IG post saying alcohol even for food (used wine to cook food)	“Alcohol on the go! Choose haha intelligent Pinoy Edit Pinoy #Covid_19PH – at Burger Matsing Bagong Silang Phase 7B [picture of a burger restaurant]”	“Because there are new COVID-19 cases now, it’s better to be safe and protected. These days, you don’t know who is sick. I’m selling Emergency Packs w/ Face Masks. There are 48 items inside. PM me for orders You can also check our FB [link to page selling packs]”	—	“How to avoid COVID and stay healthy? There’s the wearing of mask, hand washing, proper hygiene and social distancing. But aside from that, we need to BOOST our immunity by eating healthy foods and taking vitamins such as these.”
Overpositivity	“We can just laugh about nCOV, that’s how positive Filipinos are. That’s how brave we are! So easy! Filipinos will not be affected by that disease! #Ncov #Corona #KeepSafe #Afraid”	“Now I know why we are are PANICking because of COVID-19. Hehe. Because it comes from PANIC-ki [bat in Filipino] Hahahaha Kidding aside. Let us all move in faith and wisdom because God is in control. Keep safe everyone! #Covid_19“	“To fight against coronavirus. Who wants face mask?”	—	“Yes, it’s contagious but it can't destroy Juan dela Cruz [image of a person holding the Filipino flag]”
Performing credibility	“Doctor’s advice about preventing coronavirus: Wash your hands frequently and gargle with strong mouthwash to eliminate germs in your throat before entering your lungs.”	(Image showing different types of masks, including gas masks and facial masks)	“3rd day of immune system boosting against COVID-19”	“Yes- [link to a news article on the spread of coronavirus disinformation to sow panic]”	“Experts told us we can contain COVID-19 and prevent deaths. But we did not listen. We still engaged in hoarding and panic-buying. We gave in to panic and this poor lady got affected because of it.”
Political commentary	“It’s stupid. I don't think that the global panic about COVID is worth it. It’s just like any other flu. The recovery period is just longer. But you'll get better from it, especially when you don't have a health problem.”	“COVID sneaking in? [Image of Duterte coughing at presscon then gif shows a girl entering]”	—	“What if...The DOH can confirm the COVID cases ahead of time but they are just waiting for the perfect moment, like a loud issue that can stifle it, like the Chinese/POGO before they announce.”	“Are people in the government vampires? They like late announcements/briefings. One good preventive measure against the virus is a good sleep to boost your immune system. #coronavirus #nCoV19 #COVID2019”

^a^No tweets.

## Discussion

### Prevalence of COVID-19 Misinformation on Twitter

In a systematic review, health misinformation related to vaccines, drugs, and pandemics was more commonly found on Twitter than on YouTube, Facebook, and Instagram, where the more prevalent topics were noncommunicable diseases and treatments [7]. During the H1N1 pandemic, 4.5% of the manually coded tweets contained possible misinformation or speculation [15]. In this Philippine study, 6.8% of the manually coded tweets contained COVID-19 misinformation. Another study with a smaller data set and using COVID-19–related Twitter hashtags reported a higher proportion of misinformation at 24.8% [16].

The prevalence of COVID-19 misinformation on Twitter will likely vary depending on how the data set is constituted. There are 2 possible strategies to identify tweets with COVID-19 misinformation. In a top-down strategy, tweets linked to news articles that have already been fact-checked to contain misinformation are retrieved [17]. This method has the advantage of finding more tweets. However, tweets with misinformation that have not yet been fact-checked will be missed. The bottom-up strategy requires collecting tweets within a specified period and manually annotating these to identify misinformation. This has the disadvantage of finding a lesser number of tweets but has the advantage of possibly finding a wider range of misinformation, not just the more popular ones that have been fact-checked [18]. As COVID-19 misinformation can be diverse and mostly coming from ordinary people, although with less reach [14], the bottom-up strategy was used in this study.

### Characterization of Tweets With COVID-19 Misinformation

In contrast to previous literature that often used latent Dirichlet allocation (LDA) as an NLP method, this study used BTM. Tweets are short and often contain colloquial or abbreviated language, which makes automated classification difficult [19]. The LDA approach may be limited by severe data sparsity because the tweets are relatively short. BTM addresses the problem of data sparsity by modeling word co-occurrence patterns and using aggregated patterns in the corpus [20]. A study comparing the use of BTM and LDA on short texts shows that BTM can more accurately capture topics of short texts than LDA [21].

COVID-19 misinformation on Twitter was conceptualized as a phenomenon demonstrating 3 features: topics, formats, and discursive strategies. Consistent with previous evidence [7,22-29], various topics of COVID-19 information, such as nature, agents, prevention, management, and context and consequences of COVID-19, emerged from our analysis. These topics were elucidated by combining the analytic affordances of NLP and CQA of tweets and key informant interviews.

The analyses suggest that COVID-19 misinformation on Twitter assumes different formats, specifically misleading content, satire/parody, false connection, conspiracies, and false context. These formats have been demonstrated in previous COVID-19 infodemiologic research [7,14]. Methodologically, the investigation of formats in this study differs from earlier investigations in 2 aspects: the development of format subcategories was enriched by key informant interview insights, and the theoretical framework from Wardle and Derakhshan [2], which was adapted to identify these formats, was not determined a priori. Adherent to the process of constant comparative and iterative analyses, possible theoretical prepositions that can explain the patterns emerging from the data were only considered during the third stage of CCA (selective coding). Facilitating the credibility and confirmability of this categorical scheme is the CQA process: vigorous discussions were held among the members of the interdisciplinary team. Each member presented theoretical prepositions and together discussed the applicability of each until a consensus was reached to use Wardle and Derakhshan [2] as the final framework.

Compared with topics and formats, discursive strategies relied more on the subjective judgment of the research team and insights from the key informant interviews. Previous studies have attempted to examine the communicative aspects of social media posts regarding COVID-19 misinformation [30,31]. However, the linguistic features of the tweets must be drawn from Filipino discourses, cultural features, and the sociopolitical climate. Moreover, because the tweets were both in Filipino and English, implementing NLP or relying on foreign frameworks for conceptual development may lead to an inadequate analysis. Hence, a sociological eye that is sensitive to these contexts and a theorization grounded in actual data became necessary, especially because there has been no published Philippine-based research that attempted such a project. For example, an analysis of English tweets containing conspiracy theories in the early months of the pandemic identified 5G technology as spreading COVID-19 and Bill Gates patenting COVID-19 [32], but these conspiracy theories were not found in our data set.

The subcategories under discursive strategies emerged purely inductively (unlike topics that were based on BTM topic clusters and formats that were based on an accepted theoretical model). Some of the discursive strategies are demonstrations of Filipino social practices. These include humor and overpositivity, both of which are common coping mechanisms of Filipinos [33,34]. Other strategies (political commentary, anger/disgust, and fear mongering) appear to highlight the sociopolitical backdrop of the country during the start of the pandemic, which is generally medical populist in nature [35,36]. The remaining discursive strategies appear to be social practices borne out of the internet/social media culture, wherein alternative identities can easily be created (ie, performing credibility) and entrepreneurial ventures can be pursued with a lesser sense of accountability (ie, marketing).

Finally, this study offers a matrix that demonstrates the intersections of formats and discursive strategies of COVID-19 misinformation. Previous studies have noted that each type and context of COVID-19 misinformation are factors that influence the sharing and acceptance of future information regarding the pandemic [17,31,37]. This matrix can be used by governments and advocates to study misinformation patterns and design interventions to correct and combat them.

### Interdisciplinary Approach

Previous studies on detecting COVID-19 misinformation on Twitter have used qualitative methods [17,29], mostly computational [38,39], and a combination of the 2 methods [40]. This study’s methodology departs from these previous investigations by using an interdisciplinary team and incorporating key informant interviews into the data repository. The informants shared examples of COVID-19 misinformation not only on Twitter but also on Facebook, Viber, and Messenger. By deriving keywords from the interview transcripts and shared tweets, posts, and messages, which were later used in the corpus reduction process to constitute the subcorpora, we were able to triangulate COVID-19 misinformation on non-Twitter platforms and include these in the analyses. Hence, the conceptualization developed from the analyses benefits from increased confirmability while being linked to the experiences of users who were exposed to misinformation (ie, the key informants of this study) [41]. This attempt to enhance the embeddedness of web-based health misinformation behaviors in offline social contexts has been reported to be wanting in the recent body of literature [42].

### Limitations

The study has the following limitations: limited generalizability because of how the Twitter data set was constituted; limited analysis of images, links, and emojis embedded within the tweets; no sentiment analysis; and the accuracy of human manual coding.

#### Constituting the Twitter Data Set

Our Twitter data set is likely not entirely representative of COVID-19 misinformation in the Philippines. While Twitter is the fifth most popular social media platform in the Philippines, with 62.7% of internet users aged 16 to 64 years having used it in 2021, only 67% of Filipinos are on the web [43]. For households living in extreme poverty in the Philippines, television (85.5%) and radio (56.1%) were reported as the main sources of COVID-19–related information [44]. While the age distribution of Twitter users is likely not the same as that of the entire country, a greater proportion of the Philippine population is young [45], as most Twitter users are.

The corpus of tweets analyzed is not representative of all tweets from the Philippines, as the streaming Twitter API only collects up to 1% of tweets [18], and only tweets geolocated around the NCR were included. Although no Philippine-specific data are available, it was previously estimated that only 0.85% of tweets contain geolocation data, which limits the generalizability of the results [46]. Although Twitter users using smartphones can broadcast their GPS coordinates with tweets, this feature is disabled by default [47]. However, the streaming API has been shown to oversample geotagged tweets [48], which is an advantage for this study.

Only single tweets were analyzed. Retweets, quote tweets, or replies to the tweets were excluded; thus, the reach of misinformation from the collected tweets was not included in the analysis. It is possible that a tweet in the subcorpus did not contain COVID-19 misinformation, but the reply or quote tweet did. However, at least in 1 study, retweets were not significantly different for tweets with COVID-19 misinformation [16]. In addition, censorship on Twitter was not accounted for in the study. As this study dealt with retrospective data, tweets containing misinformation may have been reported as abusive or harmful and removed from the platform at the time of data collection. Tweets were also not collected from accounts marked private.

User profiles were not included in the study, as it was nearly impossible to verify the veracity of the Twitter profiles. We did not collect Twitter profile data after the enactment of Philippine Republic Act No. 11469 in March 2020, which imposed fines and imprisonment for “individuals or groups creating, perpetrating, or spreading false information regarding the COVID-19 crisis on social media and other platforms” [49]. A systematic review of health research using Twitter found that the demographics of users were reported in only 4% of cases [50].

The tweets included in the data set were not analyzed for possible bot activity, and bots can also spread misinformation [51]. However, bot presence was likely low, as retweets were excluded for this study, and bots usually retweet content without tweeting the original content [52]. An argument put forth against deleting bot tweets in a data set is that it is “artificially manipulating a raw data set,” as bots are naturally found on Twitter [53].

#### Analyzing the Twitter Data Set

While human coding is often considered the gold standard in analyzing tweets, it is not a standard classifier because language itself can be ambiguous and coder capacity changes when tired [54]. Coders who are Twitter users and are thus familiar with the “idiosyncratic linguistic conventions of tweets” [55] may perform better, although coders inherently imbue their positionality in the coding process [56]. In this study, manual coding was performed by a social scientist-nurse and a health informatician-medical specialist who have both been on Twitter for more than a decade.

Although tweets can contain links to photos and videos, some off the platform (ie, Facebook and Instagram), these were only included in the analysis if needed to understand the tweets’ context. In manually coding tweets, the researchers used the middling approach [55], where tweets were presented as textual information for coding with the option to view the tweet on Twitter if clarification was required. It is possible that tweets were manually coded without seeing linked photos or videos. A separate analysis of images attached to tweets with misinformation was not performed. A previous study found that tweets with COVID-19 misinformation images did not receive more interactions, although they were shared for longer periods and had longer burst times [40]. Emojis within tweets were also not analyzed separately. The use of emojis appeared to be gendered on Twitter during the early months of the pandemic [57].

### Conclusions

This study highlights the advantages of using an interdisciplinary, multimethod approach to understand COVID-19–related misinformation on Twitter. The integration of machine learning–aided techniques, CQA of tweets, and insights from interviews with people who are exposed to social media provides an in-depth account of the nature and potential impacts of misinformation, situated in the sociocultural context of the actors involved in the information ecosystem of interest. Through this novel method, the results reveal COVID-19 misinformation in the Philippines as a phenomenon characterizing multiple topics; assuming various formats; and using discursive strategies that are manifestations of the cultural values and norms of Filipinos in relation to disease and public crises and, hence, are unique to the Filipino experience. Aside from its methodology, this study expands the infodemiology literature by offering new conceptual frameworks to analyze health information in the country and elsewhere in Asia. The Philippines is an archipelagic nation characterized by ethnolinguistic diversity, as is the Asian continent. Thus, we recommend regional studies using the same methods to examine COVID-19 misinformation patterns across cultural subgroups. Such studies will benefit from having researchers trained in infodemic management with competencies in social listening, identification of narratives, interventions against distrust, and building resilience against infodemics [58]. The findings of this study can be used by governments and public health practitioners to design interventions to monitor, predict, and address misinformation regarding COVID-19 and other health conditions.

## Appendix

Multimedia Appendix 1 Questions used during the key informant interviews.

Multimedia Appendix 2 Emergent keywords (n=89) identified from key informant interviews.

Multimedia Appendix 3 Emergent topic clusters from biterm topic modeling (BTM) grouped according to subcategories.

## Abbreviations

API: application programming interface
BTM: biterm topic modeling
CCA: constant comparative analysis
CQA: consensual qualitative analysis
LDA: latent Dirichlet allocation
NCR: National Capital Region
NLP: natural language processing
RQ: research question
