# Supplementary material for: Interdisciplinary Approach to Identify and Characterize COVID-19 Misinformation on Twitter: Mixed Methods Study
Source: JMIR Form Res. 2023 Jun 28;7:e41134. doi: 10.2196/41134 (PMC10337476; doi:10.2196/41134)
Supplement: Multimedia Appendix 1 [file formative_v7i1e41134_app1.docx]

1. What were examples of misinformation about COVID-19 that you read circulating in facebook and twitter (and other social media)? (And then for each misinformation answered, probe using the following questions):
   1. How did you respond to this misinformation?
   2. How did others respond to this misinformation?
   3. What channels were used to spread this misinformation?
   4. What was the form of misinformation? (Narratives, pictures, memes, infographics, videos, etc.)
   5. Can you provide an example post (public domain— because of ethics)?
   6. How do you think this misinformation affects the way people see and behave towards COVID-19 and how the disease is managed?
2. How would you describe the people/accounts who:
   1. Believe COVID-19 misinformation?
   2. Shared COVID-19 misinformation?
   3. Rejected COVID-19 misinformation?
3. Can you provide public domain facebook profiles, pages, twitter accounts and sites that propagate ? (Public domain - those that can be accessed even if you are not following the account)
   1. What strategies do these public domains use in order to spread misinformation?
4. How would you differentiate the way misinformation is spread and dealt with in facebook versus twitter?
5. What efforts can be done by different sectors of society to address online COVID-19 misinformation?
