# Supplementary material for: Interdisciplinary Approach to Identify and Characterize COVID-19 Misinformation on Twitter: Mixed Methods Study
Source: JMIR Form Res. 2023 Jun 28;7:e41134. doi: 10.2196/41134 (PMC10337476; doi:10.2196/41134)
Supplement: Multimedia Appendix 2 [file formative_v7i1e41134_app2.docx]

| **Respondent number and background** | **Emergent keywords from the interview** |
| --- | --- |
| A – Pharmacist | Treat, treatment, prevent, prevention, Hydroxychloroquine, Azithromycin, CIA, military, Hong Kong, Japan, zero |
| B – Pediatrician | Chinese, China, lab, weapon, steam, tuob, cure, vaccine, family, friend |
| C - Business manager | Hot, weather, pepper, ginger, alcohol, body, heat, AIDS, tea, soup, water |
| D - Nurse and instructor | Steam, tuob, weapon, China, lab, mask, hospital |
| E - Chief Technology Officer | coconut, VCO, malunggay, banana, water, garlic, silver, Panelo, Duque, Dela Peña, UST, cocktail, Thailand, Bilibid, jail, forecast, Ebola, FASSSTER, million |
| F – Rheumatologist | university, hospital, bats, snake, capacity, friend, family, biowarfare, rash, arthritis |
| G - Pharmacist and instructor | HCQ, vitamin C, vitamin D, ascorbic, ascorbate, zinc, percent, masks, distancing, social |
| H - Physician at a health center | China, Chinese, positive, mask, white |
| I - Data management officer | Mask, white, quarantine, cure, blue, green, saging, asin, Lord, barley, acai, end, times, world |
| J – Pharmacist | SARS, MERS-CoV, AIDS, flu, coronavirus, vaccine |
