# Supplementary material for: Interdisciplinary Approach to Identify and Characterize COVID-19 Misinformation on Twitter: Mixed Methods Study
Source: JMIR Form Res. 2023 Jun 28;7:e41134. doi: 10.2196/41134 (PMC10337476; doi:10.2196/41134)
Supplement: Multimedia Appendix 3 [file formative_v7i1e41134_app3.docx]

| **Word Clusters from BTM** | **Words considered in Proposed Topics** | **Proposed Topics** | **Topic Categories** |
| --- | --- | --- | --- |
|  |  |  |  |
| safe, stay, city, spread, keep, please, lockdown, outbreak, quarantine, dont, prevent, hands, manila, affected, home, country, need, pray, chinese, news, healthy, world, hope, threat, lets, jesus, today, novel, community, metro | Safe, stay, city, spread, keep, please, lockdown, quarantine, dont, prevent, hands, affected, home, country, need, healthy, community | Safety Measures | COVID prevention and management |
| tested, travel, first, march, like, outbreak, says, year, hospital, patients, chinese, filipino, contact, city, patient, symptoms, news, system, breaking, reported, immune, filipinos, negative, novel, came, today, please, wuhan, admitted, last | test, travel, first, outbreak, hospital, patients, contact, patient, symptoms, system, reported, immune, negative, today, admitted, last | Testing |  |
| public, medical, spread, like, please, know, help, threat, novel, week, center, safety, wuhan, stop, hospital, time, think, need, chinese, masks, already, stay, thank, give, precautionary, friends, lets, family, make, measures | spread, threat, safety, stop, think, need, masks, stay, precautionary, measures | Precautions |  |
| time, mask, hands, always, dont, masks, keep, country, hygiene, immune, test, system, proper, hand, stay, maligo, kits, clean, lugar, sanitize, first, world, alcohol, think, natin, laging, really, social, cant, jakol | time, mask, hands, always, dont, masks, hygiene, immune, system, proper, hand, stay, maligo, clean, lugar, sanitize, first, alcohol, think, laging, social, jakol | Health Measures 1 |  |
| city, muna, baka, need, mayor, government, quarantine, bahay, sobrang, prevent, parang, govt, social, stress, pala, lumabas, distancing, news, lockdown, crisis, issue, mukhang, days, naka, sakin, panic, local, nako, iwas, says | Bahay, prevent, government, distancing, iwas | Adherence to Guidelines |  |
| home, dont, like, stay, please, social, spread, news, even, good, quarantine, work, know, really, mask, avoid, distancing, well, theres, risk, safety, community, stop, infected, alcohol, times, days, going, protect, today | home, dont, stay, please, social, spread, quarantine, work, mask, avoid, distancing, risk, safety, community, stop, infected, alcohol, protect | Health Measures 2 |  |
| bansa, sakit, pilipinas, duterte, like, bahay, natin, ibang, dami, baka, pinas, alam, quarantine, manila, sobrang, gobyerno, meron, please, galing, parang, puwede, wuhan, muna, lockdown, takot, taong, novel, panic, balita, kesa | like, ibang, dami, baka, alam, sobrang, meron, parang, puwede, muna, takot, novel, panic, kesa | Uncertainty | Nature of COVID |
| total, number, first, novel, country, deaths, death, source, outside, died, countries, breaking, march, patients, chinese, year, confirms, reports, safe, manila, bringing, department, wuhan, sars, world, spread, person, panic, stay, without | total, number, first, country, deaths, death, source, died, countries, breaking, patients, confirms, reports, world, spread, person | COVID Statistics |  |
| house, novel, says, duque, president, committee, measures, tested, duterte, spread, government, budget, disease, secretary, travel, quarantine, response, office, outbreak, cayetano, department, amid, said, representatives, city, montales, francisco, breaking, safe, senate | house, committee, measures, government, budget, response, office, cayetano, department, representatives, city, montales, francisco, senate | Lawmakers’ Response | People/agents of COVID |
| like, public, natin, news, testing, pala, duque, time, good, duterte, disease, government, chinese, world, said, lalo, patients, country, mass, right, media, crisis, kits, first, actually, think, panic, nasa, hands, always | news, duque, duterte, government, chinese, world, country, mass, media, crisis | International Issues |  |
| doctors, country, workers, medical, nurses, travel, please, community, work, public, wuhan, quarantine, said, hospital, lord, countries, patients, time, working, govt, police, chinese, safe, government, keep, spread, officials, allowed, nasa, needed | doctors, country, workers, medical, nurses, community, work, public, quarantine, hospital, patients, time, working, govt, police, safe, government, officials, needed | Frontliners |  |
| safe, baka, natin, guys, stay, alam, muna, niyo, pala, love, keep, chinese, dami, good, please, sure, miss, pilipinas, tayong, okay, ayaw, better, pwede, sayo, meron, travel, home, jowa, yang, tatay | safe, natin, guys, niyo, love, good, please, sure, miss, tayong, okay, ayaw, better, pwede sayo, home, jowa, tatay | Loved Ones | Contexts and Consequences of COVID |
| mask, akong, kamay, nasa, alam, natin, safe, panic, alcohol, araw, okay, muna, niyo, sakit, bang, tayong, jusko, work, time, kanina, pinas, thandoctok, center, parang, bahay, mong, yang, puwede, outbreak, ulit | mask, kamay, alam, natin, safe, panic, alcohol, muna, sakit, jusko, work, time, kanina, center, bahay, outbreak | Panic Buying |  |
| market, edge, philippine, quarantine, government, global, manila, outbreak, amid, community, president, measures, metro, cant, situation, nightly, risk, free, business, state, help, time, first, classes, shares, stocks, filipinos, says, travel, lockdown | market, edge, philippine, quarantine, global, outbreak, community, measures, situation, risk, business, state, help, time, shares, stocks, travel, lockdown | Economy |  |
| government, outbreak, manila, time, would, quarantine, work, stop, country, taal, novel, travel, cant, even, affected, last, death, dont, community, need, lets, pandemic, test, better, days, spread, symptoms, lockdown, back, kobe | outbreak. taal, kobe | Tragedies other than COVID |  |
